# The association between empathy ability and attitudes toward children with disabilities in inclusive physical education classes among primary students: the mediating role of friendship quality

**DOI:** 10.3389/fpsyg.2025.1531002

**Published:** 2025-04-17

**Authors:** Wenwei Ling, Dandan Wang, Xin Xu, Dong Zhu, Xueping Wu, Lei Zhang

**Affiliations:** ^1^School of Physical Education, Shanghai University of Sport, Shanghai, China; ^2^Department of Physical Education, University of Shanghai for Science and Technology, Shanghai, China; ^3^Department of Physical Education, Changhai County Senior High School, Liaoning, China

**Keywords:** disability attitudes, empathy, friendship quality, mediating effect, inclusive physical education

## Abstract

**Background:**

The acceptance and inclusion of children with disabilities, especially in inclusive physical education classes, is crucial for their social integration and psychological well-being. To examine the relationship between friendship quality, empathy and attitudes of children without disabilities toward their peers with disabilities in inclusive physical education classes.

**Methods:**

The Children’s Attitudes Toward Integrated Physical Education–Revised, Friendship Quality Questionnaire, and The Chinese version of the Interpersonal Reactivity Index instruments were used to investigate the attitudes of children without disabilities toward the participation in physical education classes of their peers with disabilities, the quality of their friendships, and their ability to empathize, respectively. Descriptive statistics, Pearson’s correlation analysis, linear regression, and mediation effects were used for data analysis.

**Results:**

Girls without disabilities showed more positive attitudes than boys without disabilities toward the inclusion in physical education classes of children with disabilities (total score *t* = −3.92, *p* < 0.001). Disability attitudes were significantly and positively correlated with friendship quality (*r* = 0.22, *p* < 0.001) and the ability to empathize (*r* = 0.16, *p* < 0.01). Empathy positively predicted disability attitudes (adjusted *R*^2^ = 0.055, *F* = 13.555, *p* < 0.001), and friendship quality mediated the relationship.

**Conclusion:**

Friendship quality plays a key role in mediating the effect of empathy on attitudes of children without disabilities toward their peers with disabilities. Enhancing students’ empathy indirectly improves their attitudes toward disabilities by strengthening friendships.

## 1 Introduction

Inclusive education has been a global movement since its introduction in the Salamanca Declaration (Ruijs and Peetsma, 2009). Inclusive education aims to include all students, particularly those from marginalized or excluded groups, by ensuring equal participation while addressing their emotional well-being and cognitive development (Ferguson, 2008). China has always been committed to the United Nations Convention on the Rights of Persons with Disabilities (United Nations, 2006) and formally embracing the vision of a disability-inclusive society. In China, with the incorporation of inclusive education principles into laws and policies, such as the Regulations on the Education of Persons with Disabilities (MOE, 2021), the “Learning in Regular Classrooms” (LRC) model has been expanded into a more comprehensive, quality-oriented inclusive education initiative (Deng and Poon-McBrayer, 2004; Yan et al., 2021; Xie et al., 2023). Since signing the Convention on the Rights of Persons with Disabilities on March 30, 2007, China has demonstrated a strong commitment to protecting the rights of persons with disabilities. According to the latest figures released by Ministry of Education of the Peoples Republic of China, there were 2,345 special education schools nationwide, with 265,261 students enrolled. Additionally, 462,103 students attended regular classes or special education classes in ordinary schools, accounting for 50.7% of the total, while 184,617 students with disabilities received home-based education, making up 20.2%, highlighting the significant progress of inclusive education (MOE, 2024).

When inclusive educational practices are implemented, students with disabilities who attend their neighborhood schools can receive educational services with their peers without disabilities in general education classes (Hunt and McDonnell, 2007). Similar to all curriculum areas, when considering the inclusion of students with disabilities, physical education (PE) faces many new kinds of challenges and opportunities (Qi and Ha, 2012). Numerous studies have shown that inclusive sport improves the motor skills and physical functioning of students with disabilities and their peers (Pocock and Miyahara, 2018), enhances social skills and peer relationships (Seymour et al., 2009), boosts self-esteem and psychological well-being (Goodwin and Watkinson, 2000), and promotes positive learning environments and a culture of inclusion (Pocock and Miyahara, 2018). While, achieving genuine inclusive physical education requires overcoming various challenges. Among these challenges, negative attitudes constitute a significant barrier that impedes the full societal integration and participation of individuals with disabilities. These attitudes are rooted in stereotypes, often stemming from prejudice and misconceptions portraying this population as dependent, inferior, antisocial, or incapable (Hutzler et al., 2005). It is such attitudes that can lead to the exclusion of people with disabilities from social circles. However, the positive attitudes of teachers, peers, and parents, among others, are recognized as crucial for the success of inclusive physical education. Inclusive physical attitudes and acceptance of students with disabilities can help them feel part of a group and interact positively with other students (Vaillo et al., 2016). Peer attitudes toward children with disabilities can also significantly affect their self-confidence, which in turn influences their social acceptance and even the development of a sound personality (Fu et al., 2022).

Empathy is an emotional response arising from an individual’s understanding of another person’s emotional state which consists of both cognitive and emotional components (Segal, 2018). Cognitive empathy refers to the ability to recognize and comprehend others’ emotions, whereas emotional empathy pertains to the capacity to share and resonate with others’ emotional experiences (Huang and Su, 2012). Even in the absence of direct contact, education and guidance can help children without disabilities develop an understanding of the challenges faced by their peers with disabilities in physical education classes, fostering both sympathy and empathy toward their experiences. A study found that empathy is significantly positively correlated with college students’ positive attitudes toward individuals with intellectual disabilities (Mirete et al., 2022). By understanding the feelings and thoughts of those with intellectual disabilities, college students can establish stronger connections with this group, thereby reducing social distance and enhancing inclusivity. Increased empathy not only deepens an individual’s understanding of the emotions and feelings of others but also significantly elevates the likelihood of resonating with them.

According to Batson et al. (2002), individuals simultaneously belong to multiple groups and possess diverse group identities. During intergroup interactions, competition often outweighs cooperation. However, group empathy fosters improved intergroup attitudes and promotes greater intergroup prosocial behavior. Group empathy refers to the process by which members of one group indirectly experience and internalize the perceptions, emotions, and feelings of another group, whether through direct interactions or imagined encounters (Sirin et al., 2016). Group empathy cultivates more positive attitudes toward outgroups and increases the willingness to engage in intergroup interactions (He and Xie, 2018). Moreover, contact theory posits that interaction between members of different groups can enhance intergroup attitudes, even among rival groups (West et al., 2015). Therefore, individuals are more likely to comprehend the emotional dynamics that arise during intergroup interactions and to resonate with the emotional states of outgroup members. Previous research has demonstrated that group empathy mediates the relationship between intergroup contact and attitudes toward individuals with disabilities in a study of individuals without disabilities (Armstrong et al., 2016). This finding further suggests that group empathy functions as an emotion regulation mechanism, reducing rejection of children without disabilities of their peers with disabilities and fostering the development of more positive, inclusive attitudes.

Friendship quality is another variable related to attitudes toward inclusive PE. It plays a crucial role in the social development of adolescents, particularly in promoting inclusiveness and reducing prejudice. According to Vervoort et al. (2011), high-quality majority-minority friendships are linked to reduced negative out-group attitudes and enhanced in-group attitudes. This finding underscores the significance of friendship quality in shaping adolescents’ attitudes toward various groups. When adolescents experience a deep emotional connection and mutual understanding within friendships, they are more likely to develop positive attitudes toward peers from diverse backgrounds. Additionally, as discussed in the literature, contact theory highlights the capacity of direct contact between groups to reduce prejudice under conditions of equality and cooperation (Pettigrew and Tropp, 2006). In the context of friendship, this contact transcends superficial interactions and encompasses deeper affective and cognitive processes, such as self-disclosure and the reduction of intergroup anxiety (Turner et al., 2007). Therefore, the quality of friendships not only impacts individuals’ emotional well-being but also directly influences their perceptions and acceptance of other groups. Contact theory also places particular emphasis on the importance of cross-group friendships. As a close social relationship, friendship fosters deep understanding and emotional connection, thereby helping to mitigate negative attitudes toward the other group.

In summary, both friendship quality and empathy significantly influence the attitudes of primary school children without disabilities toward the participation of students with disabilities in inclusive PE classes. Previous research has explored other influences on attitudes to participation in physical education classes among peers with disabilities, but empathy and friendship quality have not been included. Most previous studies have examined the role of friendship quality and empathic competence in disability attitudes separately. Few studies have considered the simultaneous influence of friendship quality and empathy on disability attitudes. According to the findings in a paper by Shadish, it is feasible to use mediating effects to develop and study theories of human behavior (Shadish, 1996). Therefore, the present study examined the mediating effects between empathy, friendship quality, and attitudes toward students with disabilities.

This study aimed to investigated the relationship among empathy, friendship and attitudes of children without disabilities toward their peers with disabilities in physical education classes. We hypothesized that (1) the ability of primary school children without disabilities to empathize with children with disabilities significantly predicts positive attitudes toward participation in inclusive PE classes, (2) the quality of friendships among primary school students without disabilities significantly predicts positive attitudes toward the participation of students with disabilities in inclusive PE classes, (3) the quality of friendships plays a mediating role in empathic competence on the attitudes of students with disabilities participating in inclusive PE classes. Bossaert et al. (2011) found that Belgian adolescents generally exhibited tolerant attitudes toward peers with disabilities, with gender playing a significant role. Based on this study, we hypothesize that (4) the attitudes of children without disabilities toward the participation of children with disabilities in physical education classes are influenced by gender.

## 2 Methods

### 2.1 Participants and procedures

Participants were recruited from a whole convenience sample of primary students studying at grades 3–5 in a mainstream elementary school in Shanghai (in September 2021). The initial sample consisted of 482 participants who provided informed consent. All participants were children without disabilities who had regular contact with peers with disabilities at school. During the distribution process, all students were initially allowed to complete the questionnaires. The teachers later assisted in identifying and excluding responses from students with disabilities. All participants provided written informed consent after receiving a detailed explanation of the study’s purpose, procedures, and their right to withdraw at any time without penalty. Participants were excluded from the analysis if they met any of the following criteria: having a disability according to the Chinese Classification and Grading Criteria of Disability (GB/T 26341–2010) (China, 2011), being below third grade, completing the questionnaire more than once, having more than 10% missing data, admitting to responding untruthfully or carelessly, reporting difficulties in understanding or interpreting the questions, or failing to correctly answer any instructed response items. All questionnaires were assessed for reliability and validity and demonstrated high reliability and validity. Paper-version questionnaires (described below) were distributed and uniformly administered to participants by trained psychology students, with assistance from the class teacher. Participants spent approximately 40 min completing the questionnaires anonymously, which were collected immediately afterward. The data were coded to ensure anonymity before analysis. A total of 435 valid questionnaires were obtained, resulting in an effective response rate of 90.2%. Among the valid participants (*N* = 435), there were 52.4% boys and 47.6% girls. There were 37.9% students in the third grade, 32.4% in the fourth grade, and 29.7% in the fifth grade. The mean age of the children was 10.37 years. This study was approved by the Ethics Committee of XXX (No. 102772020RT054).

### 2.2 Measures

#### 2.2.1 Peers’ attitude toward students with disabilities

The Children’s Attitudes Toward Integrated Physical Education-Revised (CAIPE-R) scale (Block, 1995) was used to investigate the attitudes of students without disabilities toward inclusive PE. The Chinese version has been validated and used in the Chinese population (Wang, 2020). The scale is composed of two subscales: general attitudes subscale, and sport-specific attitude subscale. The general attitude scale, consists of seven questions (e.g., if Jimmy were in my P.E. class, P.E. would not be fun), including two reverse-scored items. The participants rated the items on a 4-point Likert scale: no, maybe not, maybe yes, and yes. Higher scores indicated more positive attitudes toward the co-participation of students with disabilities in inclusive PE classes. The Sport-Specific Attitude Subscale consists of five items (e.g., Jimmy could have someone help him run to first base.). The participants rated the items on a 4-point Likert: no, maybe not, maybe yes and yes. Higher scores indicated more favorable attitudes toward the inclusion of a student with a disability in regular physical education. The general attitude subscale yielded a Cronbach’s alpha of 0.87 and the sport-specific subscale had a Cronbach’s alpha of 0.66.

#### 2.2.2 Friendship quality

The Friendship Quality Questionnaire (FQQ) was developed by Parker and Asher (1993) to test the quality of friendships. The scale contained 18items covering a validation and caring component (e.g., “My friend makes me feel good about my ideas”), a help and guidance component (e.g., “My friend helps me so I can get done quicker”), a companionship and recreation component (e.g., “My friend and I always sit together at lunch”), an intimate exchange component (e.g., “My friend and I always tell each other our problems”), a conflict resolution component (e.g., “My friend and I make up easily when we have a fight”), and a conflict and betrayal component (e.g., “My friend and I argue a lot”), including three reverse-scored items. The participants rated the items on a 5-point Likert: not at all true, not true, not sure, true, really true. Higher total scores indicated higher quality of children’s friendships. Cronbach’s *α* of the scale was 0.85.

#### 2.2.3 Empathy ability

The Interpersonal Reactivity Index (IRI), developed by Davis (1980) to measure empathy based on multidimensional theoretical constructs, was later introduced and revised as a Chinese version (Siu and Shek, 2005). The scale consists of four dimensions: perspective taking (e.g., “I try to look at everybody’s side of a disagreement before I make a decision.”), fantasy scale (e.g., “I often have tender, concerned feelings for people less fortunate than me.”), empathy concern (e.g., “I often have a soft heart for people in need.”), and personal distress (e.g., “I tend to lose control during emergencies.”), with a total of 22 questions. The participants rated the items on a 5-point Likert scale: not at all true, not true, not sure, true, really true. Higher scores indicated greater empathetic competence. Cronbach’s α of the scale was 0.65–0.70 (Siu and Shek, 2005).

### 2.3 Statistical analysis

IBM SPSS Statistics (version 22.0; IBM, Chicago, IL, United States) was used to conduct the statistical analyses. Data were reviewed for accuracy, missing or invalid data, distribution, and the extent to which a normal distribution was violated. Descriptive statistics were used to provide the age of the students and the scores for each scale and its subscales. Correlation analyses were conducted using SPSS to screen possible covariance issues and the correlations between the factors, and regression analyses were used to check the extent to which the independent variables predicted the dependent variables, both of which laid the groundwork for the subsequent analysis of mediation effects. The SPSS plug-in Process was used to test mediation effects, and the Bootstrap method was used to test the statistical significance. A two-side *p* < 0.05 was considered statistically significant.

## 3 Results

### 3.1 Descriptive statistics

This study examined the scores of the children (*N* = 435) across the various scales and subscales. Table 1 presents the scores, means, and standard deviations of each item. Independent samples *t*-tests revealed significant gender differences for most of the variables on the three scales. Notably, boys scored significantly lower than girls for sports-specific attitude (16.48 ± 2.49 vs. 17.30 ± 2.27, *p* < 0.001) and the total score (35.99 ± 4.69 vs. 37.77 ± 4.76, *p* < 0.001) on the disability attitude questionnaire—the CAIPE-R—as well as for intimate exchange on the FQQ (7.73 ± 2.878 vs. 9.01 ± 2.666, *p* < 0.001). Furthermore, except for conflict and betrayal on the FQQ, the *t*-values for all other variables assessed in the three scales were less than 0, indicating that girls performed better on each scale.

**Table 1:** Descriptive and inferential statistics for each variable (*N* = 435).

Variables	Boys (*N* = 228)	Girls (*N* = 207)	*t*	*p-*value
Age (years)	10.36 ± 0.93	10.38 ± 1.07	−0.203	0.840
CAIPE-R (score)				
General attitude	19.50 ± 3.58	20.49 ± 3.39	−2.940	0.004
Sports-specific attitude	16.48 ± 2.49	17.30 ± 2.27	−3.540	<0.001
Total score	35.99 ± 4.69	37.77 ± 4.76	−3.920	<0.001
IRI-C (score)				
Perspective taking	13.57 ± 4.05	14.14 ± 3.87	−1.497	0.135
Personal distress	9.22 ± 4.89	10.84 ± 4.78	−3.481	0.001
Fantasy scale	12.93 ± 4.11	13.54 ± 3.76	−1.603	0.110
Empathy concern	13.54 ± 3.39	14.08 ± 3.50	−1.628	0.104
Total score	48.76 ± 11.26	52.07 ± 10.22	−3.199	0.001
FQQ (score)				
Validation and caring	8.27 ± 2.60	8.78 ± 2.49	−2.085	0.038
Help and guidance	8.38 ± 3.09	9.31 ± 2.69	−3.326	0.001
Companionship and recreation	9.01 ± 2.59	9.68 ± 2.60	−2.699	0.007
Intimate exchange	7.73 ± 2.88	9.01 ± 2.67	−4.787	<0.001
Conflict resolution	8.49 ± 2.95	9.09 ± 2.72	−2.199	0.028
Conflict and betrayal	8.54 ± 3.52	8.35 ± 3.26	0.574	0.567
Total score	50.41 ± 12.51	54.21 ± 12.13	−3.210	0.001

CAIPR-R, Children’s Attitudes Toward Integrated Physical Education – Revised; IRI-C, The Chinese version of the Interpersonal Reactivity Index; FQQ, Friendship Quality Questionnaire.

### 3.2 Correlation analysis

**Figure 1:**
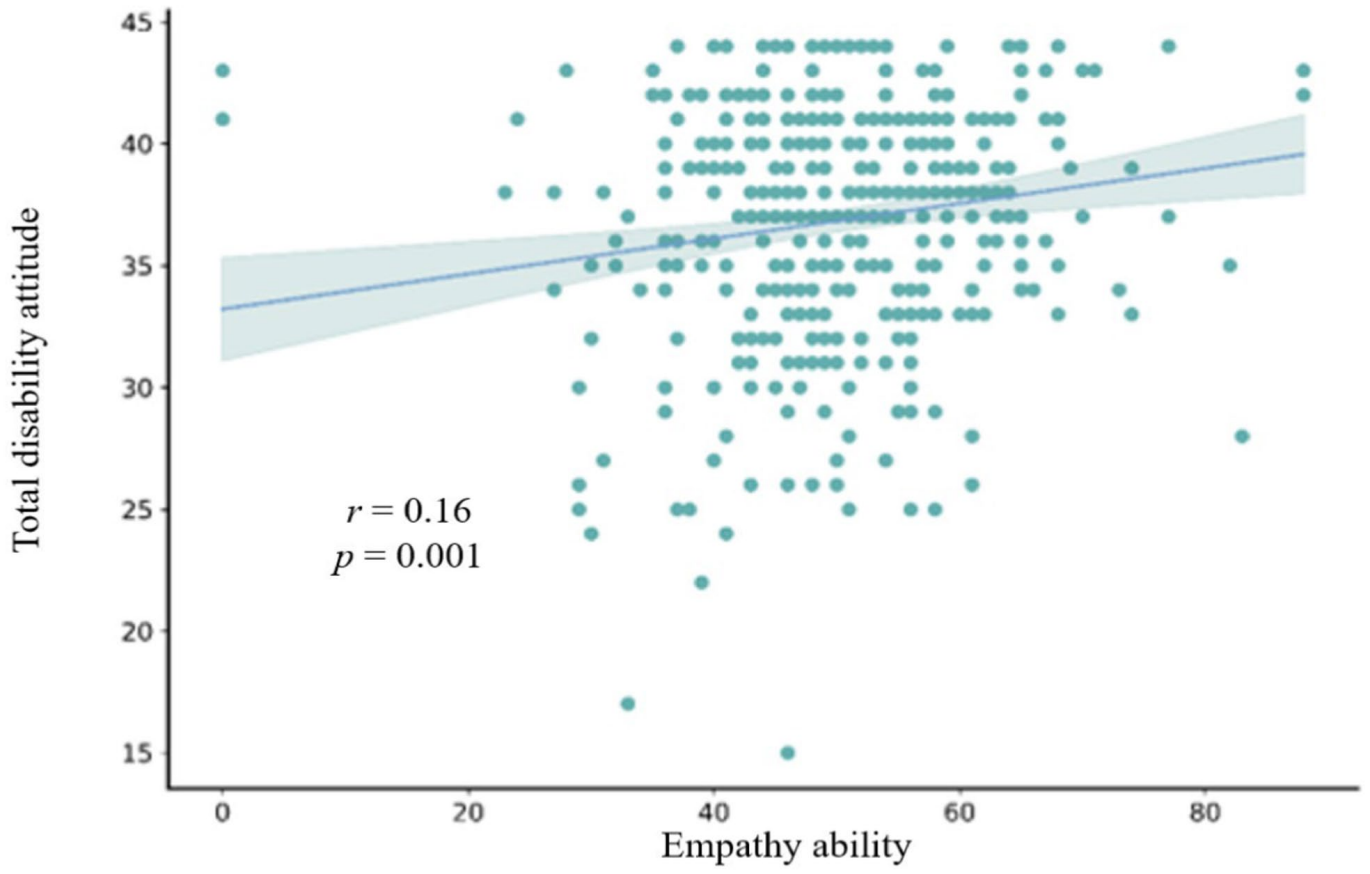
Scatterplot of correlation between the total empathy score and the total disability attitude score.

**Figure 2:**
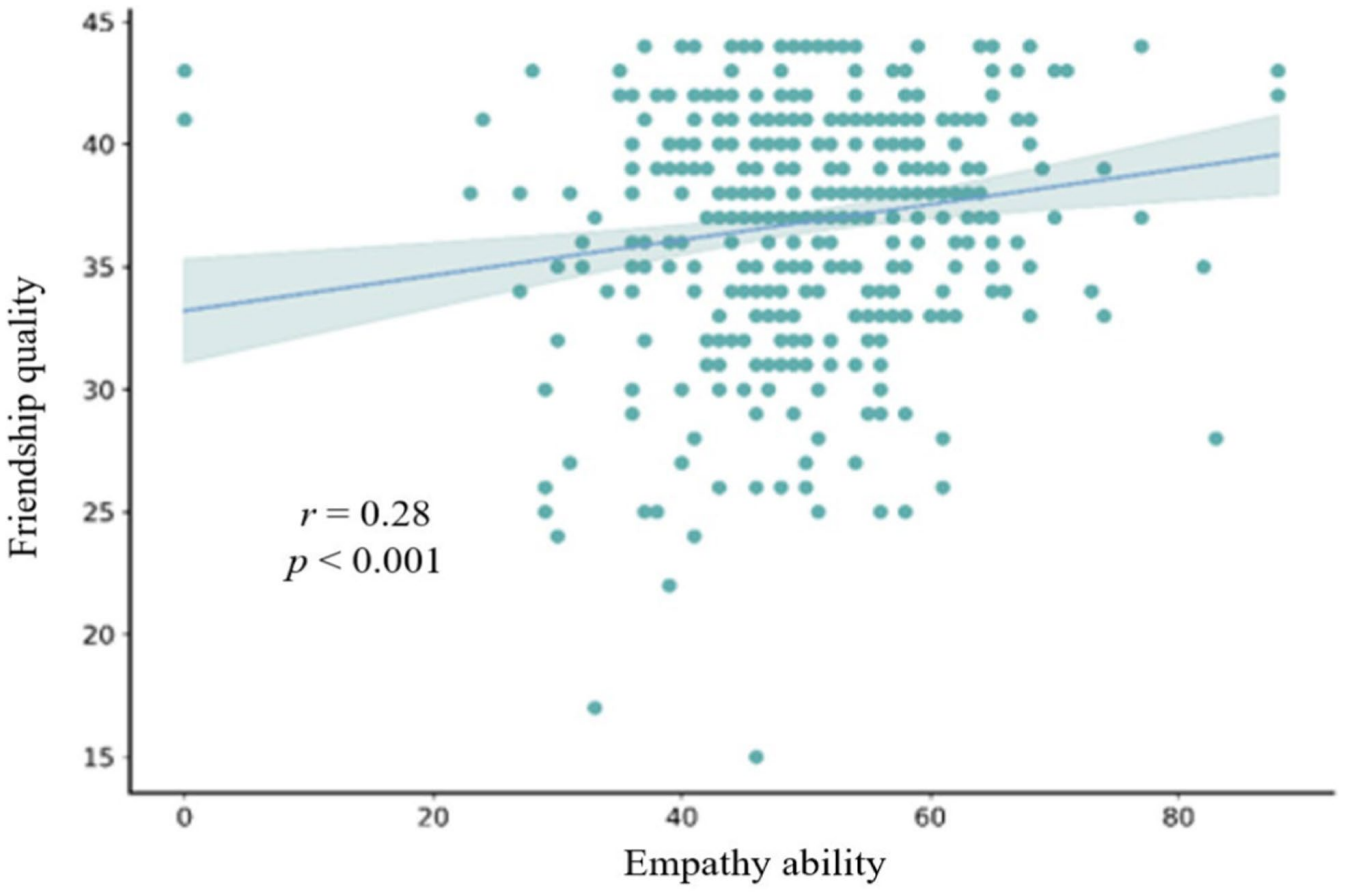
Scatterplot of the correlation between the total empathy score and the total friendship quality score.

**Figure 3:**
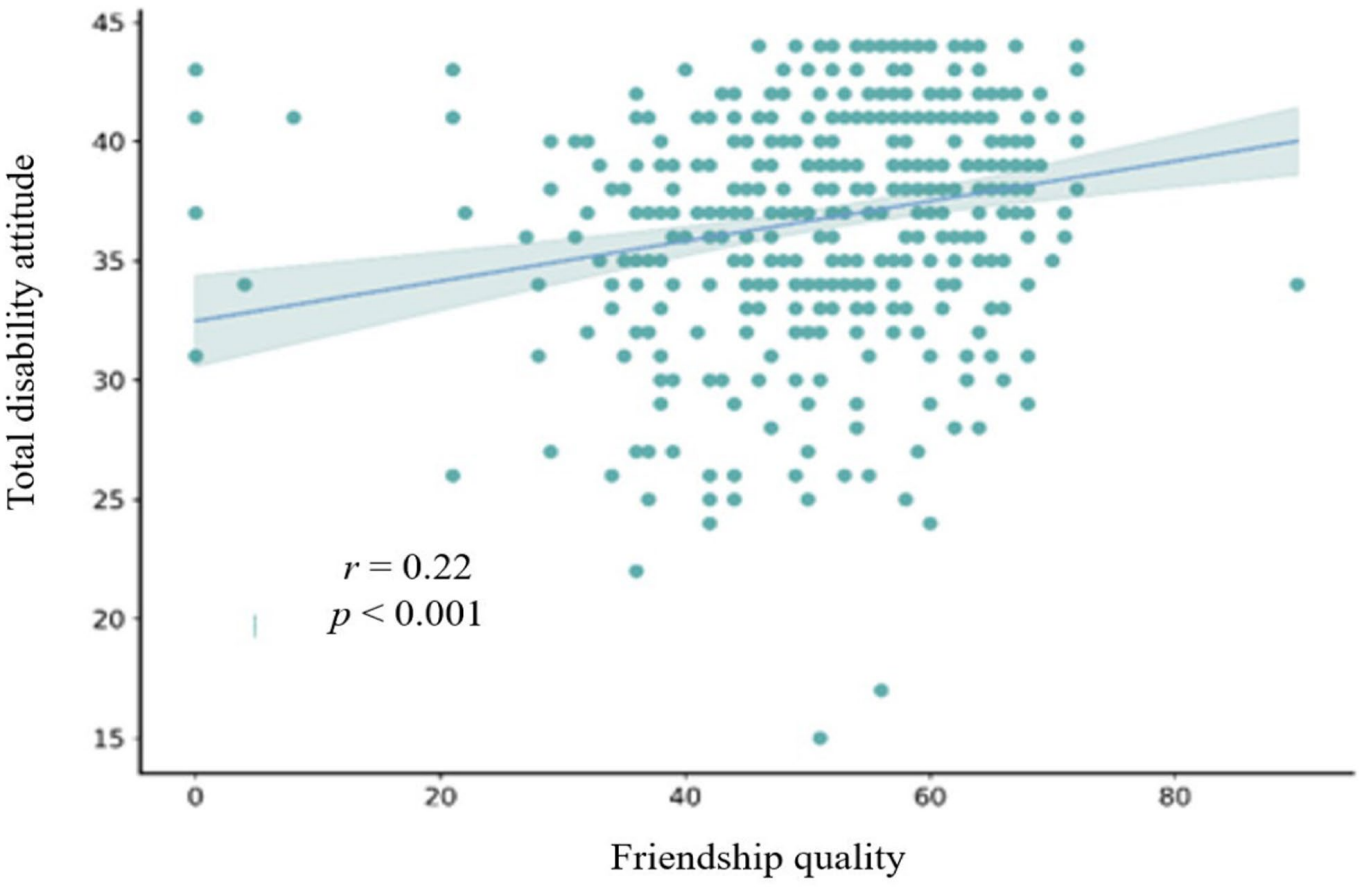
Scatterplot of the correlation between the total friendship quality score and the total disability attitude score.

This study also explored the relationships among all variables. To achieve this, Pearson correlations were performed for empathetic competence, friendship quality, and disability attitude. The correlation statistics presented in Table 2 indicate that the statistical significance levels between sports-specific attitudes, disability attitude, friendship quality, and general attitude were all below 0.01. A scatter plot of the correlation between empathetic competence and disability attitude is shown in Figure 1. It shows a slope greater than 0, indicating a significant correlation between empathy and disability attitude (*r* = 0.16, *p* < 0.01). Similarly, significant correlations were observed between empathy and friendship quality (*r* = 0.28, *p* < 0.001), as well as between friendship quality and disability attitude (*r* = 0.22, *p* < 0.001). These correlations are depicted in Figures 2, 3. Pearson correlation analysis also identified significant positive relationships between empathy and disability attitude [*r*(433) = 0.16, *p* < 0.01], suggesting that children without disabilities and with higher levels of empathy tended to have more favorable attitudes toward individuals with disabilities. Furthermore, friendship quality was significantly and positively correlated with disability attitude [*r*(433) = 0.28, *p* < 0.001], suggesting that children without disabilities and with higher quality friendships were more likely to exhibit positive attitudes toward individuals with disabilities.

**Table 2:** Descriptive statistics and correlation matrix between variables (*N* = 435).

Variables	Mean score	Standard error	1	2	3	4
Correlation matrix						
1. General attitude	19.97	3.52				
2. Sports-specific attitude	16.87	2.42	0.28^***^			
3. Disability attitude	36.83	4.80	0.88^***^	0.71^***^		
4. Empathy	50.33	10.89	0.14^**^	0.12^**^	0.16^**^	
5. Friendship quality	52.22	12.46	0.20^***^	0.14^**^	0.22^***^	0.28^***^

^**^p < 0.01, ^***^p < 0.001.

### 3.3 Linear regression analysis

To examine the effects of empathy and friendship quality on disability attitudes, linear regression analyses were conducted, using empathy and friendship quality as predictor variables and the dimensions of the CAIPE-R scale as outcome variables. As is shown in Tables 3, empathy and friendship quality were significant positive predictors of the three dimensions of disability attitudes (*F* = 13.555, *p* < 0.001). Empathy (*β* = 0.112, *p* = 0.022) and friendship quality (β = 0.187, *p* < 0.001) both had significant positive effects on disability attitudes. Together, they accounted for 5.50% of the total variance in disability attitudes. Additionally, the test for multicollinearity revealed that the variance inflation factor values were all below 5, indicating no multicollinearity problem.

**Table 3:** Results of regression analyses of empathy ability and friendship quality scores on attitudes toward disability (*N* = 435).

Dependent variables	Predictor variables	R^2^	Adjust R^2^	*F*	*SE*	*β*	*t*	*p*-value
General attitude score	Empathy	0.047	0.043	10.63***	0.016	0.089	1.822	0.069
	Friendship quality				0.014	0.174	3.562	<0.001
Sports-specific attitude score	Empathy	0.028	0.023	6.149**	0.011	0.092	1.852	0.065
	Friendship quality				0.010	0.116	2.339	0.020
Total attitude score	Empathy	0.059	0.055	13.555***	0.021	0.112	2.292	0.022
	Friendship quality				0.019	0.187	3.844	<0.001

^**^p < 0.01, ^***^p < 0.001.

### 3.4 Intermediation effect analysis

**Figure 4:**
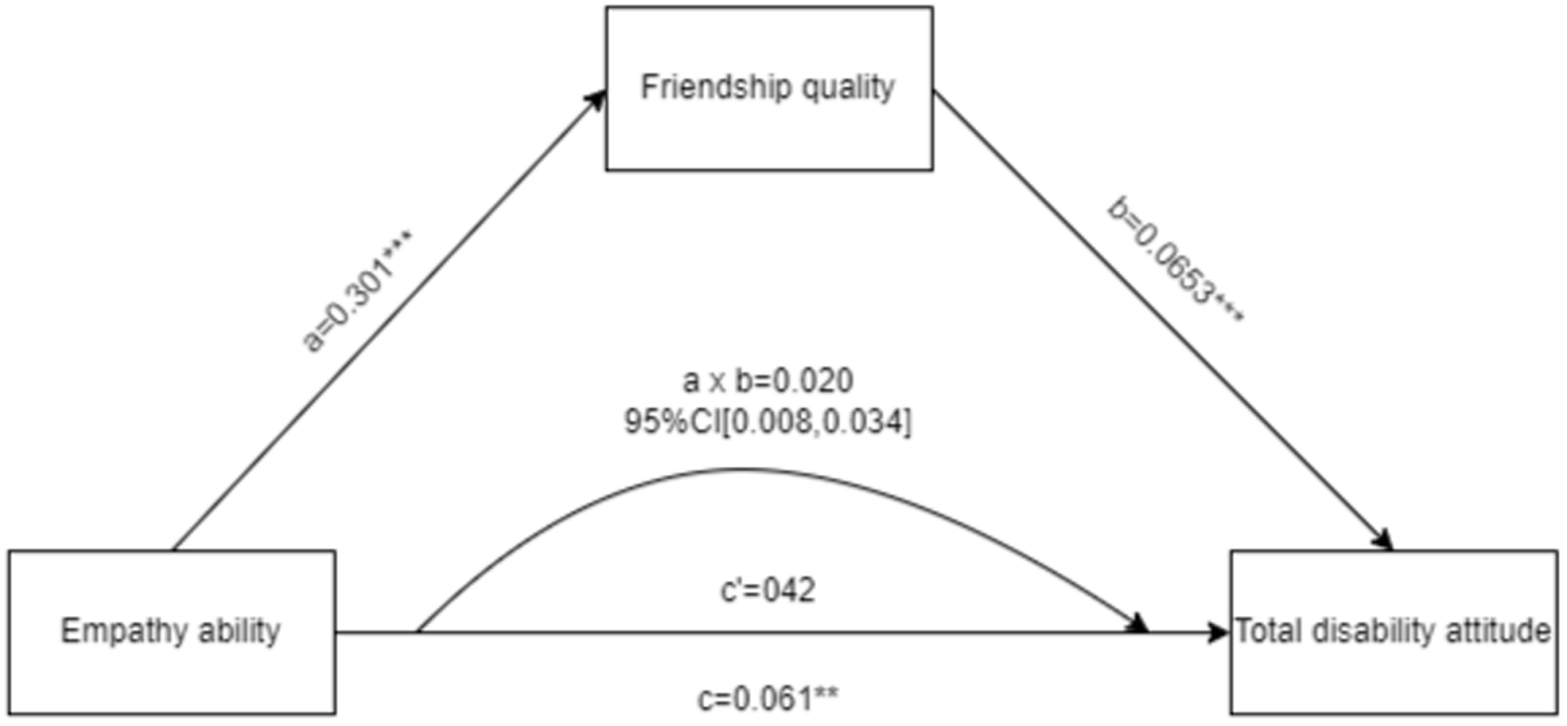
The effect of empathy on disability attitudes: the mediating role of friendship quality. Values above the pathways indicate the results of the mediation analysis, with a × b representing the indirect effect; c, the total effect; and c’, the direct effect. ^**^ *p* < 0.01, ^***^p<0.001.

Based on the results of the correlation analyses, this study further examined whether friendship quality mediated the relationship between empathy and attitudes toward disability. The mediation model, with gender as a control factor, empathy as the predictor, attitudes toward disability as the outcome, and friendship quality as the mediator, is presented in Figure 4. The results showed that (1) the total effect was significant (*p* = 0.0035), indicating the presence of a mediating effect, and (2) the indirect effect was significant (95% CI [0.008, 0.0342]), while (3) the direct effect was not significant (95% CI [−0.0004, 0.0836]), indicating full mediation. These findings suggested that friendship quality fully mediated the relationship between empathy and attitudes toward disability.

## 4 Discussion

Numerous studies have examined primary school students’ attitudes toward peers with disabilities, focusing on the influence of schools and teachers. Teachers’ language, behavior, attitudes toward inclusivity, and implementation of inclusive education practices play a crucial role in shaping students’ acceptance of peers with disabilities (Avramidis and Norwich, 2002). Similarly, an inclusive school environment provides more opportunities for positive interactions and collaboration, thereby reducing stereotypes and fostering a deeper sense of equality and understanding (Fu et al., 2022). Taking an individual perspective and including all disability categories, this study examined the relationship between the empathetic competency of primary school children without disabilities and their attitudes toward students with disabilities participating in inclusive PE classes, and the mediating role of friendship quality in this relationship.

The results indicated that empathetic competency was significantly and positively correlated with attitudes toward students with disabilities participating in PE, meaning that higher empathy levels were associated with greater positive attitudes toward the participation of these students. Although no research has previously directly examined this relationship, classical social psychological theory suggests that individuals are more likely to act favorably toward others when they feel strong empathy (Batson, 2011). Previous research has demonstrated that empathy is a psychological process occurring in contexts of intergroup interactions, such as social stigma, prejudice, and stereotyping. Experimental studies on empathy, which involves putting oneself in another’s shoes, have confirmed that empathy can serve as a mechanism to reduce negative intergroup relations (Batson and Ahmad, 2010). The success of compassion training methods such as reflective practice (Sansó et al., 2015), experiential learning methods (Sinclair et al., 2020), and others confirms that compassion can be activated and enhanced through an individual’s reflection and learning (Klimecki et al., 2014). In PE classes, students with disabilities, who lag behind their typically developed peers in motor development, fitness, and sports participation due to physical limitations, often face challenges in participation and are susceptible to discrimination and negative treatment by their peers (Wu and Zhang, 2013). In this context, students without disabilities who are highly empathetic are more likely to accept and include students with disabilities in PE classes. Therefore, the results of the present study suggest that empathetic competency in primary school students can be considered an important moderator of specific social relationships. Enhancing the ability of students without disabilities to understand others’ emotions and feelings, and increasing opportunities to learn about students with disabilities, can help eliminate inherent prejudices and change the attitudes of students without disabilities toward students with disabilities.

The results of the present study also indicated that the quality of primary school children’s friendships positively predicted their attitudes toward the participation of students with disabilities in PE classes. Although few studies have examined the relationship between friendship quality and primary school children’s attitudes toward students with disabilities, previous research suggests that friendship quality represents the closeness of a friendship between two individuals. Children who are more accepting of their peers in social interactions tend to have better interaction skills, are more likely to form and maintain friendships, and thus tend to have higher friendship quality (Chow et al., 2013). Research has shown that the attitudes of children without disabilities toward peers with disabilities are more negative than toward peers without disabilities and that girls tend to have more positive attitudes than boys (Bossaert et al., 2011). These differences may be due to variations in peer interaction levels and individual backgrounds. Besides teacher attitudes, class size, and type of disability, which are important factors influencing the attitudes of children without disabilities toward students with disabilities in an integrated education setting, lower friendship quality can lead to negative social attitudes among children (De Boer et al., 2012). These children may be unaccepted by their peers, have sparse friendships, and feel lonely, which can result in exclusionary or bullying attitudes toward students with disabilities (Laws and Kelly, 2005). Additionally, research suggests that children with better self-awareness have more open attitudes toward their peers with disabilities. Children who have extensive contact or selective relationships with peers with disabilities are likely to be more aware of, and sensitive to, their peers’ disabilities, showing more positive attitudes (Schwab, 2017). Therefore, the significance of findings of the present study is that creating empathetic situations can enhance high-quality interactions between students and their peers. This can increase the awareness of students without disabilities toward their peers with disabilities, thereby changing their attitudes in the PE classroom.

This study also found that greater empathetic competence among primary school students is positively related to friendship quality and can positively predict it, consistent with previous research. Numerous studies have confirmed that empathy plays a crucial role in forming good interpersonal relationships. It helps maintain healthy relationships and improves friendship quality (Grühn et al., 2008). Individuals with high levels of empathy are more sensitive to others’ feelings and needs and tend to have more friends (Peter et al., 2017). The mediation effect test results of the present study showed that the empathetic competence of primary school students without disabilities not only directly affected attitudes toward the participation of peers with disabilities in PE but also indirectly affected these attitudes through the mediation of friendship quality. In the context of sports, positive friendships and peer interactions have been found to lead to a stronger motivation to participate and a more positive perceptions of sports in the PE classroom (Weiss and Smith, 2002). Students with high levels of empathy tend to have more positive peer interactions in PE classes, as their ability to understand others’ feelings enables them to have significant emotional experiences. Therefore, the participation of children both with and without disabilities in sports is recognized as an important means of reducing prejudice, discrimination, and social exclusion among students without disabilities toward students with disabilities (Raiola, 2015). For primary school children, those with greater empathetic competence are able to participate more positively and optimistically in group activities. This generates higher-quality peer relationships and friendships, which in turn manifest in more positive and inclusive attitudes toward students with disabilities in PE classes.

Our analyses of gender differences showed that girls scored significantly higher than boys in empathy, friendship quality, and attitudes toward students with disabilities. Some researchers suggest that females exhibit more pro-social behaviors than males because they are generally more empathetic and more likely to feel compassion in situations requiring help (Loke et al., 2011). This is closely related to females being better than males at experiencing and expressing empathy (Lietz, 2010), among other socio-cognitive and affective functions. Additionally, previous research has found that gender is the most common determinant of attitudes of children without disabilities toward peers with disabilities, with females tending to show more positive attitudes than males (Vignes et al., 2009). Thus, females are more likely to engage in pro-social behaviors driven by empathy, reflecting a higher level of empathy, a greater ability to understand others’ feelings, more satisfying friendships, and more positive attitudes toward students with disabilities during their development and socialization (Cordellieri et al., 2020).

## 5 Limitations

Our study explored the impact of primary school students’ empathy and friendship quality on their attitudes toward participation in PE classes with peers with disabilities from an individual perspective. There are some limitations to this study. Firstly, the participants were all from urban areas, and primary school students in rural areas were not surveyed. Therefore, the findings may not be generalizable to rural areas or to other cities with different demographics. Future research should expand the sample size and explore the attitudes of children from different regions, economic levels, and family backgrounds toward the participation of students with disabilities in PE classes. This will help determine if the relationship between empathetic competency and friendship quality differs from the results of this study. Secondly, this study did not investigate the school environment or teachers’ attitudes, which may have influenced the results. Future research should consider the potential impact of the school environment, including teachers’ attitudes, on children’s attitudes. Finally, some studies suggest that the type of disability is an important factor in children’s attitudes toward their peers with disabilities, and that different types of disability can make children have different attitudes (Fu et al., 2022). However, because the subjects in this study had limited experience with or knowledge of children with different disability categories, and because our research focused on examining primary school students’ attitudes toward peers with all disability categories, we did not differentiate between specific disability types. Future research should explore this aspect to examine whether the relationship between the attitudes of students without disabilities toward the participation in PE of their peers with disabilities and the qualities of empathy and friendships are affected by the type of disability.

## 6 Conclusion and implications

This study found that the attitudes of primary school students without disabilities toward the participation in PE of their peers with disabilities were positively related to the empathetic competency of students without disabilities and the quality of their friendships. Empathetic competency not only directly influenced disability attitudes but also indirectly influenced them through the mediating role of friendship quality. The study results suggest that integrating empathy training with peer interaction activities may be a potential means to positively influence the attitudes of students without disabilities toward the participation in PE classes of students with disabilities. Future research could further explore how children’s empathetic competency and friendship quality can be effectively enhanced through education and interventions, and how these psychological traits influence other social attitudes and behaviors.

## Data Availability

The raw data supporting the conclusions of this article will be made available by the authors, without undue reservation.
